# Analysis of genetic biomarkers, polymorphisms in ADME-related genes and their impact on pharmacotherapy for prostate cancer

**DOI:** 10.1186/s12935-023-03084-5

**Published:** 2023-10-19

**Authors:** Khurram Rehman, Zoya Iqbal, Deng Zhiqin, Hina Ayub, Naseem Saba, Muzammil Ahamd Khan, Liang Yujie, Li Duan

**Affiliations:** 1 Faculty of Pharmacy, Gomal University, D.I.Khan, Pakistan; 2 Department of Orthopedics, The First Affiliated Hospital of Shenzhen University, Second People’s Hospital, ShenzhenShenzhen, 518035 Guangdong China; 3 Guangdong Provincial Research Center for Artificial Intelligence and Digital Orthopedic Technology, Shenzhen Second People’s Hospital, Shenzhen, 518035 Guangdong China; 4 Department of Gynae, Gomal Medical College, D.I.Khan, Pakistan; 5 Institute of Biochemistry and Biotechnology, D.I.Khan, Pakistan; 6 Department of Child and Adolescent Psychiatry, Shenzhen Kangning Hospital, Shenzhen Mental Health Center, Shenzhen, 518035 Guangdong China

**Keywords:** SNPs, Androgen metabolism, Pharmacotherapy, Biomarkers, Prostate cancer, Genetic polymorphism

## Abstract

Prostate cancer (PCa) is a non-cutaneous malignancy in males with wide variation in incidence rates across the globe. It is the second most reported cause of cancer death. Its etiology may have been linked to genetic polymorphisms, which are not only dominating cause of malignancy casualties but also exerts significant effects on pharmacotherapy outcomes. Although many therapeutic options are available, but suitable candidates identified by useful biomarkers can exhibit maximum therapeutic efficacy. The single-nucleotide polymorphisms (SNPs) reported in androgen receptor signaling genes influence the effectiveness of androgen receptor pathway inhibitors and androgen deprivation therapy. Furthermore, SNPs located in genes involved in transport, drug metabolism, and efflux pumps also influence the efficacy of pharmacotherapy. Hence, SNPs biomarkers provide the basis for individualized pharmacotherapy. The pharmacotherapeutic options for PCa include hormonal therapy, chemotherapy (Docetaxel, Mitoxantrone, Cabazitaxel, and Estramustine, etc.), and radiotherapy. Here, we overview the impact of SNPs reported in various genes on the pharmacotherapy for PCa and evaluate current genetic biomarkers with an emphasis on early diagnosis and individualized treatment strategy in PCa.

## Introduction

Prostate cancer is a frequently diagnosed malignancy, estimated 1.3 million newly diagnosed cases worldwide annually. It has surpassed breast cancer and become the most prevalent, increasingly crucial medical issue in males. Among 10 million clinically diagnosed PCa men, approximately 0.7 million are living with metastatic PCa, and more than 0.4 million deaths occur annually. This mortality rate is expected to double by 2040 [1]. Despite improvements in metastatic PCa treatment managing this disease remains challenging [2]. Prostate cancer cells are increasingly resistant to various treatments, which can affect the course of the disease and survival [3]. The mortality rate will be high if the development of resistance continues to outpace the development of new treatment options. Physicians can evaluate the chance of PCa recovery using different types of statistics called survival statistics [4]. According to the survival rate statistic, only a percentage of patients survive cancer [5, 6]. Since 2014, incidence rates for prostate cancer in its advanced stages have increased by 5% annually. Overall incidence rates have increased by 3% annually [7–9]. The above finding is not surprising due to the limited resources for prostate cancer screening and detection [10–12].

Almost 98% of PCa cases originated from the organ's glandular part, and their microscopic examination is based on certain glandular patterns. The Gleason score is a commonly used assessment technique to grade prostate adenocarcinoma and has remarkable prognostic value [13]. Most malignancies arise in the peripheral glandular zone, which results in asymptomatic prostatic cancer at earlier stages, whereas symptomatic presentation occurs at the metastatic state of the disease [14]. Despite advanced ages, suggestive evidence provided by family history data reported that the critical risk factors for PCa are genetic factors that may lead to the progression of abnormal prostatic cell growth and are responsible for developing cancerous cells [15]. The initial emergence of PCa in the majority of men population is due to hereditary factors, having a family member’s history, and the chance of its occurrence in first-rank relatives is increased by two to three-fold [16]. However, the findings of segregation analysis of multi-case families supported an autosomal dominant inheritance mode, but it is estimated that this inherited form causes only 9% of all PCa. A multigenic etiology has also been proposed for the majority of PCa cases. In intraepithelial neoplasia lesions, the multilayered luminal epithelium is observed, which serves as a promising biomarker of adenocarcinoma, such as loss of cytokeratin-5 and cytokeratin-14 (basal markers), the gain of cytokeratin-8 and cytokeratin-18 (luminal markers), and altered expression of α-methyl acyl-CoA racemase [17].

In current clinical practice, inadequate diagnostic investigations are involved in screening PCa patients that are usually based on blood prostate-specific antigen (PSA) levels and the tumor stage. The classification of tumor stages is based on the blood PSA level, progression of PCa, and Gleason score of tumor grading. Though PSA is a commonly used diagnostic and prognostic marker of PCa, but numerous studies highlighted their poor correlation with survival outcomes [18]. For early prediction and prognosis of PCa, recent studies published evidence focused on the clinical importance of a genetic feature called Single Nucleotide polymorphisms (SNPs). Single nucleotide polymorphism (SNP) is the substitution, insertion, or deletion of a single nucleotide at a specific genomic position. It is the most prevalent type of genetic variation in people. A single base pair difference in the DNA sequence at a specific location in the genome causes the difference. SNPs may affect several aspects of an individual's biology, including disease susceptibility, drug response, and phenotypic traits [19]. Many SNPs in the human genome appear roughly every 300 nucleotides [20]. Specific SNPs also impact susceptibility to disease and treatment response. For instance, a specific SNP may increase an individual's risk of developing a specific disease or alter the response to a specific drug. These SNPs associated with certain traits or diseases are identified through genome-wide association studies (GWAS) [21]. Researchers identified phenotypic-related genetic markers by comparing SNP profiles of patients with healthy controls. The function of genes relating to particular pathways is altered by genetic variations that may have significant implications in clinical practice for personalized medicine [22].

These studies have evaluated the coding sequences and assessed long noncoding RNAs (LncRNAs) having more than 200 nucleotides. Although LncRNAa does not translate, they interact with DNA, RNA, and proteins to perform their regulatory effects for differentiating, migrating, and proliferating cells and inducing apoptosis [23]. A polymorphism in the promoter region of LncRNA also modulates the expression pattern. Recently, a *GAS5* gene encodes tumor suppressor LncRNA (Growth arrest-specific 5) reported to be involved in developing many cancers, such as lung, prostate, colorectal, and breast [24]. *GAS5* is considered to cause the invasion, proliferation, migration, and metastasis of PCa cells, but its exact expression level is still controversial [25]. Numerous studies highlighted that the various genetic polymorphisms are linked with the risk level, grading, and mortality of PCa. In the promoter region of *GAS5*, a 5-bp indel polymorphism is reported as variant rs145204276, shown as “-/AGGCA”, alters the gene expression pattern, which results in increased susceptibility to cancers. This SNP also significantly affects prognosis, disease stage, and the Gleason score of PCa [26].

An oncogenic transcription factor (*TMPRSS2* and *ERG* fusion) is the most frequently reported chromosomal aberration in PCa, which causes carcinogenesis in > 50% of patients. In the prostate tumor-permissive inflammatory microenvironment, epithelial transformation is followed by a phenotypic and genotypic series of changes [27]. Up till now, about 5000 somatic mutations have been detected in prostate growth, and among these, the highly reported mutated genes are *MED12*, *SCN11A, CDKN1B, SPOP, PIK3CA, PTEN, THSD7B, C14orf49, NIPA2, TP53, FOXA1*, and *ZNF595*. Almost 15–25% risk of PCa is found in individuals having mutations in the *BRCA* gene, and life-threatening prostate cancer is reported to be linked with the mutations in *BRCA1, BRCA2,* and *HOXB13* [28].

Recent molecular genetic studies on the pathogenesis of the tumor, including the inactivation of tumor suppressor genes and activation of oncogenes, have explained the multiple genetic alterations. The loss of heterozygosity causes chromosomal instability that inactivates the tumor suppressor genes, which can serve as an indicator to identify these genes containing chromosomal regions for selective growth and are found as the primary source of tumorigenesis [29]. The high-frequency loss of heterozygosity is a form of allelic loss observed in tumor suppressor genes located on chromosomes 16q and 10q and are involved in the pathogenesis of human PCa [30]. As an alternative to curative PCa therapy, active surveillance (measuring cancer progression) is a strategy for monitoring old-age patients when their low life expectancy is anticipated. However, there is a very high chance of PCa diagnosis at an older age. A reduction of 46% in mortality risk has been observed in older men treated with local therapy compared to patients treated conservatively [31]. This review presents an overview of the influence of the SNPs reported in different genes on the pharmacotherapy for PCa and assesses present genetic biomarkers with a focus on early diagnosis and personalized therapeutic approach in PCa.

## Prostate cancer biology

There are three human prostate structural zones; central, transition, and peripheral. Mainly prostate tumors arise in the outermost peripheral zone, either with luminal or basal cancer-initiating epithelial cells, which give rise to lesions indicative of adenocarcinomas [32–34]. The PCa oncogenesis is linked with a series of interactions between various factors, including somatic acquired genetic mutations, germline susceptibility, macro-environment, and microenvironment [35–37]. Tumors are complex tissues of multiple distinct cell types that undergo collaborative interactions during tumorigenesis. To maintain the tumor growth, invasion, or metastasis, the tumor cells are highly selective to shape their microenvironment by allowing the critical supportive interaction among tumor cells via soluble factors and extracellular matrix (ECM) [38]. The multiple foci forms in localized prostate cancer have many genetic alterations, diverse metastatic seeding capacities, and inherent resistance to the treatment. It is also well established that prostate carcinogenesis is also promoted by urinary microbes-induced chronic inflammation and infections, which leads to the generation of oxidative stress by free radicals to damage the DNA [39]. The proliferative inflammatory atrophy increases the number of proliferative luminal epithelial cells in the prostate, which are highly susceptible to epigenetic and genomic chromatin alterations that initiate malignant transformation and intraepithelial neoplasia [40].

**Fig. 1:**
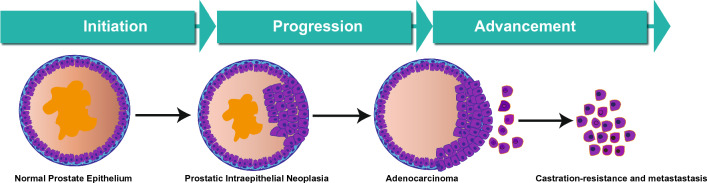
PCA initiation, progression, and advancement

There are several diagnostic tests to determine PCa staging, including prostate-specific antigen (PSA) blood tests, a digital rectal exam, imaging tests, and biopsies [41]. The specific stage of PCa plays a crucial role in determining treatment options and prognosis. PCa is characterized by three main terms: initiation, progression, and advancement. Imitation occurs when normal prostate gland cells are genetically mutated to cause PCa's development [42]. The combination of genetic predisposition and environmental factors can cause these mutations. Although the exact cause of PCa initiation is still unknown and is being studied, reported risk factors include age, family history, race, and certain genetic abnormalities [43]. PCa that occurs in distant parts of the body is called metastatic PCa. At this stage, treatment options may include hormone therapy, chemotherapy, targeted therapies, immunotherapy, and participation in clinical trials [44]. Figure 1 shows the different stages of PCA.

### Prostatic intraepithelial neoplasia (PIN)

A premalignant condition of epithelial cells that occurs due to neoplastic growth in benign prostatic acini or ducts is called prostatic intraepithelial neoplasia. The reduction or loss of basal epithelium by hyper-proliferation of luminal epithelial cells is linked with a malignancy precursor called prostatic intraepithelial neoplasia [45]. Transformation into a malignant tumor has multiple steps, such as intraepithelial neoplasia origination, localized PCa followed by advanced adenocarcinoma, and culmination with metastatic cancer [46]. A Gleason grading system defined by Donald Gleason is now widely used in clinical settings to grade the aggressiveness of prostate cancers. The prostatic intraepithelial neoplasia can be categorized as high or low grade based on the extent of intraepithelial neoplasia [47]. The prostatic intraepithelial neoplasia is considered a high grade if lesions are produced by the multilayered luminal epithelium, which can serve as transformation-related biomarkers, such as the absence of basal markers [(KRT5), (KRT14) and TP63], gaining of luminal markers [(KRT18) and (KRT8)], and overexpression of α-methylacyl-CoA racemase (AMACR). The most common chromosomal aberration is an oncogenic transcription factor resulting from the fusion of TMPRSS2 and ERG genes [48].

### Metastatic prostate cancer

In metastatic cancer, invasion of tumor cells occurs in surrounding tissues where they undergo a series of inter and intracellular complex remodeling process, which has been classified into five stages. Each stage is highly energy-demanding for the cancer cells [49]. Prostate cancer-associated mortality generally causes by a metastatic disease, which primarily metastases in the primary tumor adjacent lymph nodes, followed by the lungs, liver, and bone cancer. Bone metastases produce osteoblastic lesions that cause bone pain, frequent fractures, and hypercalcemia [50]. Among other cancers, Epithelial-mesenchymal transition (EMT) has been reported to be involved in the metastasis of prostate cancer cells by disseminating as circulating tumor cells (CTCs) into systemic circulation that easily crosses physical barriers to develop bone metastasis [51].

In a mechanistic design study, molecular and phenotypic characteristics of CTCs were focused on understanding the dissemination of cancer cells to distant organs and detecting novel prognostic biomarkers. It was observed that the metastatic tumor cell invasion of the bones is caused by stromal cell-derived factor-1 (SDF-1) and its receptor (CXCR4) [52]. SDF-1 anchor, Annexin A2, directs the binding of hematopoietic stem cells to the niche to enhance expression levels for proliferation in prostate cancer cells and apoptosis resistance during patient chemotherapy [53]. Bone metastasis is a major clinical condition of PCa. Previous studies showed a comparison between non-metastatic and progressive castration-resistance human samples and reported that more than 80% of bone lesions were found in all men who die with PCa, and the mechanism behind the prevalence of PCa in bone is not well understood yet. However, the highest mortality rate was found in the patients diagnosed with skeletal metastasis [54]. In PCa-induced mortality, Ras and other GTP-binding proteins perform several important cellular functions, such as intracellular signaling and cytoskeletal assembly. Ras is a glycosylated transmembrane protein that acts as a membrane transducer and regulates the various downstream cellular events such as proliferation, apoptosis, and invasion [55]. The Ras family consists of h-*ras*, k-*ras*, m-*ras*, n-*ras*, and r-*ras,* associated with 30% of solid tumors. As the tumor load grows, the invasion of malignant cells also upsurges in the systemic circulation. The dissemination of Latrogenic cells occurs during clinical procedures such as prostate biopsy, transurethral resection of the prostate (TURP), and brachytherapy [56].

A prostate biopsy involves the removal of small samples of tissue from the prostate gland using a needle. It is generally safe to perform this procedure; however, there is a small risk that the cells may be displaced and spread to other body parts, such as the bloodstream or nearby tissues [57]. The risk of significant complications from a prostate biopsy is relatively low. However, factors such as the needle traversing different areas of the prostate and possible bleeding at the biopsy site can contribute to cell dissemination during a biopsy [58]. Transurethral prostate resection (TURP) is a surgical procedure used to treat benign prostatic hyperplasia (BPH) by removing excess prostate tissue through the urethra with a resectoscope. Although the procedure aims to remove prostate tissue, iatrogenic cell dissemination is possible, particularly if the procedure involves cutting or manipulating tissue near the prostate [59]. The possibility of iatrogenic cell dissemination exists in both cases. Healthcare professionals must take the appropriate precautions during these procedures to minimize complications and risks and monitor patients for adverse reactions [60]. Previous studies explained that tumor growth is linked with the cellular clearance process of the circulation, which may take almost 4 weeks. This cellular clearance is mainly related to the arrest of cellular clumps in the first capillary and other factors affecting the cellular motility of differential PCa cells and differences in the chemo-attraction [61].

### Castration-resistant prostate cancer (CRPC) and ADT

**Fig. 2:**
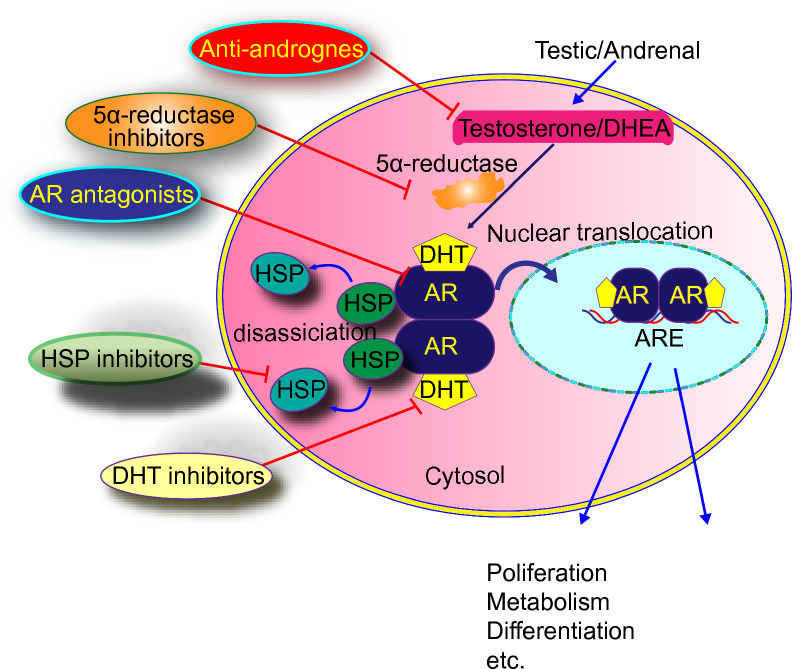
Signal transmission through AR is the main pathway for prostate cancer cell growth and spread. Therefore, regulating androgen receptors (ARs) in cells is the key to many cancer-related genes. Testosterone or dehydroepiandrosterone is converted to dehydroepiandrosterone by 5 α reductase. DHT then dissociates HSP and AR to form a complex, which is transferred to the nucleus and activates cancer-associated genes. Androgens and androgen receptors (AR) in cells regulate cancer-related genes. It is possible to control human androgen-related malignant tumors by targeting androgen signaling pathways in tumor cells with anti-androgen, 5-α reductase inhibitors, heat shock protein 90 inhibitors, androgen receptor agonists, and serotonin inhibitors

PCa progresses despite androgen deprivation therapy (ADT) or hormone therapy known as castration-resistant prostate cancer (CRPC). It is the primary treatment for advanced prostate cancer and reduces male hormones, specifically testosterone. These hormones fuel prostate cancer cell growth [62]. It is established that ADT is a cornerstone in PCa management, both as a primary treatment and in combination with other treatments. ADT can be achieved by using a variety of approaches, such as surgical castration (the removal of the testicles) or medical castration (the use of medications that suppress testosterone production [63]. The application of ADT initially controls the growth and spread of prostate cancer, which leads to the shrinkage of tumors and the relief of symptoms, but in some cases, the cancer cells continue to grow despite low testosterone levels and lead to the development of CRPC [64]. ADT alleviates symptoms by reducing cancer cell growth in locally advanced and metastatic PCa conditions. It can also be used as adjuvant therapy to eliminate residual cancer cells after primary treatment to reduce the risk of disease recurrence. This is done in combination with other treatments. In cases where PCa has spread to other body parts, ADT can improve symptoms, such as bone pain, by shrinking the tumors and decreasing their activity [65]. It is a vital component of palliative care and integral to improving the quality of life for patients with advanced disease. Along with other targeted therapies, such as abiraterone acetate or enzalutamide, ADT can further suppress androgen signaling pathways and inhibit cancer cell growth. These combinations of treatments have improved CRPC outcomes. Castration-resistant prostate cancer cells develop mechanisms to survive and grow despite low testosterone levels. These mechanisms include mutations in the androgen receptor, an increase in androgen synthesis, the amplification of the androgen receptor, and activating alternative signaling pathways [66] (Fig. 2).

## Genetic biomarkers for early prostate cancer detection

Numerous cancer research studies have validated tumor-associated genetic aberration-based biomarkers, which can help predict the risks, early diagnosis, and prediction of therapeutic outcomes. An aggressive tumor cannot be distinguished only by biopsy and blood PSA tests [79]. The dysregulation of LncRNAs controls the critical cancer hallmarks that can serve as an attractive biomarker for diagnosing PCa. Several cancer-specific LncRNAs are upregulated in PCa, such as PCATs, PCA3, SPRY4-IT1, SChLAP1, and TRPM2-AS. The altered expression pattern of LncRNA promotes the progression of tumors and metastasis [80]. Several investigative studies on determining prostate cancer antigen 3 (PCA3) level in urine have confirmed the specificity and sensitivity of this non-invasive test [81]. Noncoding RNAs (ncRNAs) have gained significant importance in tumor biology and can potentially act as cancer biomarkers. PCAT-1 is a prostate cancer-associated ncRNA transcript that acts as a prostate-specific regulator for cancer cell proliferation and is a suitable PCa marker [82]. α-methyl acyl-CoA racemase (AMACR) is a mitochondrial and peroxisomal enzyme overexpressed in prostate cancer, while its low expression level was observed in benign prostatic tissue. Hence, AMACR is a promising prostate tumor marker for early diagnosis [83] (Table 1).

**Table 1:** Types and functions of genetic markers of prostate cancer

Genetic Biomarkers	Marker types	Sample types	Functions	References
SChLAP1 (lncRNAs)	RNA	Plasma	lncRNAs regulate epigenetic modification and transcription by modulating histone or DNA	[96]
PCA3	Prostate cancer antigen 3	Urine	This gene is prostate-specific noncoding mRNA, it is assessed in a urine sample to determine the stage, grading, and aggressiveness of PCa	[97]
PCAT-1	RNA Gene	Blood	PCAT-1 is involved in cancer. It regulates proliferation, cell cycle, apoptosis, metastasis, DNA repair, and homologous recombination	[98]
AMACR	Gene	Blood	This gene encodes an enzyme called **α**-methylacyl-CoA racemase (AMACR)	[99]
CDKN2A	Gene	Tissue	Cyclin-dependent kinase inhibitor 2A located at chromosome 9	[100]
GOLPH2	cis-Golgi-localised protein	Tissue	In more than 90% of cases, Golgi membrane antigen is produced by an overexpressed gene	[101]
TMPRSS2-ERG	Prostate-specific and androgen-response gene	Urine sample	TMPRSS2 is a serine protease encoding prostate-specific and androgen-responsive gene involved in prostate carcinogenesis, and the ERG gene encodes a protein that serves as a transcriptional regulator	[102]
CCND2	Cell cycle regulatory gene	Tissue	This gene encodes proteins belonging to the cyclin family	[103]
PIM1	Gene	Blood	PIM1 is a protein kinase-encoding gene. Its expression level is significantly high in advanced prostate cancer cases	[104]
PTEN	Gene	Blood	The gene encodes an enzyme found in almost all tissues	[105]
p14b	Gene	Urine	Methylation of this tumor suppressor gene link with the degree of malignancy	[106]
PDLIM4	A protein coding gene	Whole blood	Reduced expression levels of PDLIM4 occur by hypermethylation which is helpful in the detection of prostate tumorigenesis	[107]
NKX3A	The protein encoded by the NKX3-1 gene	Whole blood	Transcription factor helps in the development of prostate epithelium. Mutation or losses in this gene may lead to the development of prostate cancer	[108]
GSTP1 Hypermethylation	An isozyme encoded by the GST pi gene	Urine	Hypermethylation inactivates the GSTP1, which plays a role in liver cancer	[109]
*GSTP-1* (Glutathione S-transferase P1)	Ubiquitous enzymes cause detoxification	Whole blood	A tumor suppressor in PCa	[110]
RB1	Gene	Whole blood	In advanced cancer stages, allelic loss or mutation leads to loss of function of tumor suppressor	[111]
CNA of Genome	Copy number variations (CNVs)	Whole blood	CNVs in the specific genomic regions in somatic cells	[112]
TP53	Gene	Whole blood	In advanced cancer stages, allelic loss or mutation leads to loss of function of tumor suppressor	[113]
ASC/TMS1 (PYCARD)	An adaptor protein activating caspase-1	Plasma	The immune response regulator encoded by this gene and its hypermethylation is found in 40% of cases	[114]
*ASPM, ASF1B, BUB1B, BIRC5, CENPF, CDC20, CDCA8, CDC2, CDCA3, CDKN3, DLGAP5, DTL, CEP55,C18orf24,FOXM1, PLK1, MCM10, NUSAP1, KIF20A, KIAA0101, PRC1, RRM2, PBK, TOP2A, TK1, RAD51, RAD54L, PTTG1, CENPM, and ORC6L*	Genes	Whole blood	Genes to predict metastatic risk and treatment outcomes	[115]
EPB41L3	A protein coding gene	Whole blood	This gene encodes a cortical cytoskeleton protein in more than 70% of prostate cancer cases	[116]
CpG islands	Genomic regions containing a large number of CpG dinucleotide repeats	Whole blood	Progression and development of PCa occur due to hypermethylation in these regions, disrupting the normal function of various genes	[117]
*APC, GSTP1 or GSTP1, RASSF1A, RARB2, MDR1*	Genes	Whole blood	Combined hypermethylation assays can determine benign and cancerous alterations in the prostate	[118]
RASSF1A	Gene	Serum	In benign prostate, hypermethylation is observed in the gene's promoter region. While in the promoter region, a patchy pattern of hypermethylation is indicative of carcinomas	[119]
RNASEL	Gene	Whole blood	DNA hypomethylation acts as the hallmark of the hereditary prostate cancer gene	[120]
TNFSR10D/DCR2	Gene	Whole blood	Gene down-regulates in PCa by hypermethylation. This gene encodes DR4 and DR5 receptors of the intracellular death domain (DD)	[121]
Polycomb components (PcG proteins)	Transcriptional repressor	Whole blood	Increased expression of polycomb complexes and chromatin modification may reveal prostate cancer's progression	[121]
HDAC1	Gene	Whole blood	TMPRSS2-ERG gene fusion caused by a histone deacetylase is involved in prostate cancer	[122]
DLC1	Gene	Whole blood	Methylation leads to gene repression that occurs extensively in prostates of older men; it may be the marker for early-stage prostate cancer	[123]
LINE-1 retrotransposons	Class I transposable elements in DNA	Whole blood	In metastatic cases, hypomethylation occurs in these sequences, while hypermethylated retrotransposons are observed in normal conditions	[124]
CDKN1C	Gene	Whole blood	Hypermethylation causes the inactivation of the gene in prostate cancer	[125]
*Ki-67*	Nuclear protein	Whole blood	It is associated with Cell-cycle-proliferation and is a predictive marker for PCa	[126]
*PSCA*	Prostate Stem Cell Antigen	Whole blood	Increased PSCA expression linked with capsular invasion in prostate cancer	[127]
IGF2	Gene	Whole blood	The IGF2 gene encodes for insulin-like growth factor 2, which controls the growth and division of cells. Differential methylation loss before manifesting carcinomas and methylation change in IGF2 is a pre-neoplastic condition in the prostate	[128]
*MME*	Membrane metalloendopeptidase	Whole blood	Biomarker linked with the progression of PCa	[129]
H3K4	DNA packaging protein Histone H3	Whole blood	The poor prognosis of PCa is linked with increased dimethylation at lysine residue	[130]
H3K18	DNA packaging protein Histone H3	Plasma	The poor prognosis of PCa is also linked with increased acetylation activation of the marker	[131]
JMJD3	Histone demethylase	Cell/tissue extract	The overexpression of demethylase is found in metastatic prostate cancer	[132]

*lncRNAs* Long non-coding RNAs, *AMACR* Alpha-methylacyl-CoA Racemase, *PCA3* Prostate Cancer Antigen-3, *PCAT-1* Prostate Cancer Associated Transcript-1, *GOLPH2* Golgi Membrane Protein-1, *TMPRSS2-ERG* Transcriptional Regulator Erg-Transmembrane Protease Serine 2, *PIM1* Proto-Oncogene Serine/Threonine-Protein kinase, *PTEN* Phosphatase and Tensin Homolog, *DTL* Denticleless E3 Ubiquitin Protein Ligase Homolog, *NKX3A* omeobox protein NKX-3, *GSTP1* Glutathione S-Transferase Pi Gene, *RB1* Retinoblastoma Protein 1, *CNA of Genome* Copy Number Alteration of Genome, *TP53* Tumor Protein p53, *ASPM* Assembly Factor for Spindle Microtubules, *ASF1B* Anti-Silencing Function 1B Histone Chaperone, *BUB1B* BUB1 Mitotic Checkpoint Serine/Threonine Kinase B, *ASC* Apoptosis-Associated Speck-like Protein Containing a CARD, *TMS1* Target of Methylation-Induced Silencing, *BIRC5* Baculoviral IAP Repeat Containing 5, *CENPF* Centromere Protein F, *CDC2* Cyclin Dependent Kinase 1, *CDCA3* Cell Division Cycle Associated 3, CDCA8 Cell Division Cycle Associated 8,*CDKN3* Cyclin Dependent Kinase Inhibitor 3, *CDC20* Cell Division Cycle 20, *DLGAP5* DLG Associated Protein 5, *CEP55* Centrosomal Protein 55, *C18orf24* Spindle And Kinetochore-Associated Protein 1, *FOXM1* Forkhead Box M1, PLK1 Polo like Kinase 1, MCM10 Minichromosome Maintenance 10 Replication Initiation Factor, *PRC1* Protein Regulator Of Cytokinesis 1,* KIAA0101 *PCNA Clamp Associated Factor,* RRM2* Ribonucleoside-Diphosphate Reductase Subunit M2*, TOP2A* DNA Topoisomerase II Alpha, *NUSAP1* Nucleolar and Spindle Associated Protein 1,* KIF20A* Kinesin Family Member 20A, *TK1* Thymidine kinase 1,* RAD51* RAD51 Recombinase,* RAD54L, PTTG1* Pituitary Tumor-Transforming Gene 1,* CENPM* Centromere Protein M, *EPB41L3* Erythrocyte Membrane Protein Band 4.1 Like 3, *RASSF1A* Ras Association Domain Family 1 Isoform A, *RARB2* Retinoic Acid Receptor B2,* APC* Adenomatous Polyposis Coli,* GSTP1* Glutathione S-Transferase Pi 1, *MDR1* Multi Drug Resistance, RNASEL Ribonuclease L, *TNFSR10D* Human Tumor Necrosis Factor Receptor Superfamily, Member 10D, *DCR2* Decoy Receptor 2, *PcG proteins* Polycomb Components, *HDAC1* Histone Deacetylase 1, *DLC1* Deleted in Liver Cancer 1, *CDKN1C* Cyclin Dependent Kinase Inhibitor 1C, *IGF2* Insulin Like Growth Factor 2, *H3K4* Histone H3 lysine K4, *JMJD3* Jumonji domain-containing 3

PCa markers are also assessed in urine samples that are a favorable alternative to serum-based biomarkers. Golgi phosphoprotein-2, a Golgi membrane antigen encoded by *GOLPH2,* is reported to be overexpressed in almost 90% of PCa patients, it does not only serve as a suitable biomarker for early diagnosis but also helps in distinguishing normal cells from cancerous cells [84]. The present studies have successfully established the relationship between aggressive PCa phenotype and TMPRSS2-ERG fusion because the overexpression of the TMPRSS2-ERG gene is linked with shorter survival of PCa patients. It also possesses prognostic significance as a tumor cell marker [85]. A protein kinase encoding the *PIM1* gene is not expressed in the benign prostatic epithelium, but its expression level is elevated significantly in advanced PCa cases. Therefore PIM1 is a promising target for developing *PIM1* inhibitor drugs [86]. Another useful prognostic biomarker is PTEN, a tumor suppressor usually deleted in prostate cancer and independently linked with the risk of lethal prostate cancer progression [87]. Hypermethylation of the *PDLIM4* gene is also used as a marker for cancer detection because, in prostate cancerous cells, up-regulation of the expression level of mRNA of PDLIM4 and its protein was found, and it acts as a tumor suppressor [88] (Table 1).

In multiple cancers, hypermethylation has been observed to indicate the earliest somatic genome alterations. Several studies on cancer have also highlighted the aberrant methylation patterns at specific genes. The hypermethylation at *GSTP1* is used to detect PCa and has been correlated significantly with the tumor stage. It also allows the early detection of more than 82% of PCa [89]. Several tumor suppressors (PTEN, RB1, and TP53) undergo mutations or allelic loss in an advanced stage of PCa, while the rare mutations are found in the RAS family (proto-oncogenes) [90]. After the surgical procedure, the DNA copy number alteration (CNA) burden across the genome of PCa patients is linked with metastasis. The CNA burden is an independent prostate-specific biomarker with a significant prognostic impact in conservative treatment [91]. The initial localized prostate cancer management is very complex. Three commonly available commercial tests (the Cell Cycle Progression score, the Genomic Prostate score, and Genomic Classifier) provide the maximum supporting information to manage and treat localized prostate cancer. Among the prognostic markers, 12 genes-based prostate markers help clinicians in the early diagnosis of PCa [92]. For appropriate assessment of pharmacotherapy, robust prognostic markers can be used, which are based on the altered expression pattern of 31 reported genes (*ASPM, ASF1B, BUB1B, BIRC5, CENPF, CDC20, CDCA8, CDC2, CDCA3, CDKN3, CEP55, C18orf24, DLGAP5, DTL, FOXM1, PLK1, MCM10, NUSAP1, KIF11, KIF20A, KIAA0101, PRC1, RRM2, PBK, TOP2A, TK1, RAD51, RAD54L, PTTG1, CENPM,* and *ORC6L*) [93]. Hypermethylation in CpG islands of DNA of cancer tissues is used as a diagnostic marker of prostate cancer. DNA methylation occurs at the specific promoter of CpG islands that can cause gene repression, such as in the case of GSTP-1 and DAB2IP, while DNA hypermethylation does not occur in normal cells [94]. In the postoperative setting, biochemical recurrence causes the progression of the disease, which can lead to lethal prostate cancer. Several studies have explained that metastasis development after biochemical recurrence is linked with the validated differential gene expression that can be used as metastatic biomarkers [95] (Table 1).

## Association between single nucleotide polymorphisms (snps) and prostate cancer

The inter-individual germline DNA differences are called genetic polymorphisms, which are differences in genomic sequences that occur between individuals at the frequency of about 1% of the general population. The most commonly reported polymorphisms in the repeated sequences (microsatellites) are Single-nucleotide polymorphisms (SNPs) [67]. A genome base pair variation in the DNA sequence is called SNP, with a frequency of about 1 out of 800 base pairs. These SNPs induce clinically significant changes in cellular proteins and enzymatic machinery. Several studies suggested that SNPs are not only important in the inheritance of genes within families but exert a strong influence on the susceptibility or risk of prostate cancer in certain individuals than others [68]. It has been reported that the entire human genome contains almost 2 million SNPs which are classified based on their functions into the following types; promoter regions SNPs are called regulatory SNPs (rSNPs); A SNP region at which nucleotide substitution causes the substitution of amino acid/ affects a protein is called as coding SNP (cSNP). Silent SNPs (sSNPs) are not involved in amino acid substitution and are present in the exon region; in the intronic region, (iSNPs) are located; and intergenic regions SNPs are called genome SNPs (gSNPs) (Fig. 1) [69].

SNPs are also responsible for altering the gene expression pattern and protein function. Regulatory SNPs and amino acid-substituting SNPs cause differences in the functional and phenotypic traits, respectively. Moreover, the gene expression level is also affected significantly by sSNPs and iSNPs [70]. The rearrangements that occur in the genomic structure or copy number alterations are usually involved in the early development of prostate cancer, however, SNPs are less commonly involved in the early development of PCa. In 40–60% of early prostate cancer patients, genomic aberrations are observed, such as fusion in TMPRSS2-ERG, whereas 5–15% of patients exhibited loss of function mutation in *SPOP* genes, and 3–5% of patients showed gain-of-function mutations in *FOXA1*. The androgen receptor gene (AR) alterations are also rarely observed in early prostate cancer [71]. Most prostate tumors in Asian men are caused by recurrent hotspot mutations in *CHD1, FOXA1,* and *ZNF292*, while only a few cases showed TMPRSS2-ERG fusions. In localized PCa, few deletions in *PTEN* and mutations in the *TP53* gene have been detected, and the frequency of occurrence of these deletions increases in patients with advanced disease states [72]. It has been well established by fine-mapping and genome-wide association studies (GWAS) that the susceptibility of PCa is associated with more than 100 commonly reported SNPs. The 8q24 polymorphisms are reportedly strongly linked with prostate cancer susceptibility, representing a promising biological marker for diagnosis and pharmacotherapy [73]. The variants of genes involved in oxidative stress, steroid metabolism, angiogenesis, cell adhesion, DNA repair, and cell cycle can also serve as suitable candidates for the disease state. An association analysis has reported 63 susceptibility loci for PCa in more than 140,000 men [74].

Previously, the clinical diagnosis was based on the digital examination or detection of blood levels of prostate-specific antigen (PSA) for prostate cancer screening. While the risk of PCa progression was also co-related to PSA, tumor stage, and Gleason score [75]. Recently, many studies highlighted the importance of SNPs and genomic alterations in the prediction, prognosis, and outcomes of pharmacotherapy of PCa. Apart from the coding sequence's role, tumor biology has assessed the effects of almost 200 nucleotides long noncoding RNAs (LncRNAs) in the development of PCa, which do not translate into proteins [76]. By interacting with macromolecules, LncRNAs perform several important cellular regulatory functions such as differentiation, migration, proliferation, and apoptosis [77]. The LncRNA promoter region containing genetic variants modulates gene expression patterns of methylation. Recently, the *GAS5* gene encoded LncRNA termed Growth arrest-specific 5 (GAS5) was found to act as a tumor suppressor in prostate, breast, lung, and colorectal cancers. It is considered that *GAS5* may be involved in the migration, invasion, proliferation, and metastasis of PCa cells; however, the exact *GAS5* expression level is still controversial in PCa cells [78]. Several studies have evidence that the down-regulation of microRNA-21/microRNA-1284 and up-regulation of PTEN/ PCDC4/AKT are linked with the expression of *GAS5* to induce apoptosis and reduce the proliferation rate of prostate cancer cells (Fig. 1) [47]. Figure 2 represents the functions and locations of SNP genes in translated and untranslated regions.

## Pharmacogenomics and pharmacogenetics of prostate cancer

The phenotypic variations occur due to an alteration of expression level or activity in the corresponding genes, which are not only linked with vulnerability to disease but also significantly affect pharmacotherapy outcomes [133]. Pharmacogenetics is the variability in drug response due to heredity or polymorphism in a single gene. This term is used to study the genes involved in the metabolism of drugs, whereas ‘Pharmacogenomics’ is the study of all genes in the DNA, which may help determine the drug's response [134]. Irinotecan treats prostate cancer by exerting cytotoxic effects by 7-ethyl-10-hydroxycamptothecin (SN-38), an active metabolite. The irinotecan-induced toxicity is associated with the polymorphisms in the genes involved in the irinotecan metabolic pathway. A polymorphism in the UDP-glucuronosyltransferase encoding gene UGT1A1 (UGT1A1*28 and UGT1A1*6) causes a decrease in metabolic enzyme activities, which results in delay in SN-38 metabolism and a higher incidence rate of adverse events [135]. Pharmacotherapy is linked with genetic background and interactions with several factors, such as acquired somatic genetic alterations, inherent germline susceptibility, and micro-environmental (immune cells) and macro-environmental (blood, lymph vessels) conditions [136]. For PCa patients, excessive advancement has been made in the therapeutic landscape of pharmacotherapy, such as Androgen-deprivation therapy (ADT) which is considered the gold standard for the primary pharmacotherapy of PCa [137]. Recently, second-generation anti-androgen agents have been developed for castration-resistant prostate cancer (CRPC) patients, such as CYP17 inhibitor abiraterone, apalutamide, enzalutamide, and darolutamide [138]. These drugs are also used for hormone-sensitive prostate cancer (HSPC) patients; however, initially, these were developed for treating CRPC patients. Nowadays, various therapeutic options are available for CRPC and HSPC patients. Among taxane chemotherapy, docetaxel and cabazitaxel have been used for CRPC treatment, whereas docetaxel is used to treat HSPC [139]. Also, there is a need to identify useful, suitable candidates for maximum efficacy and individualized pharmacotherapy regimen. The aberrant activation of androgen receptor signaling pathways (AR) is linked with castrate-resistant prostate cancer. Therefore, polymorphisms in AR pathway-related genes significantly impact the therapeutic efficacy of primary androgen deprivation therapy (PADT) by influencing the AR signaling activity [140]. The SNPs of various other genes have also significantly impacted the therapeutic outcome of primary ADT for the treatment of prostate cancer, as shown in (Table 2).

**Table 2:** Types of genetic polymorphisms (SNPs) in various drugs-related genes associated with the outcome of pharmacotherapy of prostate cancer patients

Gene Name	Function of gene	rs number	Type of polymorphism	Chromosome	Therapeutic agents	References
CYP17A1	Metabolism of androgen	rs6162	sSNP	10	Abiraterone acetate	[152]
		rs743572	rSNP			[153]
CYP19A1	Metabolism of androgen	rs1870050	iSNP	15	Anastrozole, Letrozole	[154]
		rs4775936				
CYP1B1	Encodes drug metabolizing enzyme	rs1056836	cSNP	2	Docetaxel	[155]
HSD3B1	Metabolism of androgen	rs1047303	cSNP	1	Abiraterone acetate	[156]
		rs1856888	gSNP			[142]
HSD17B2	Metabolism of androgen	rs4243229	iSNP	16	Enzalutamide	[152]
		rs7201637				
HSD17B3	Metabolism of androgen	rs2257157	iSNP	9	Abiraterone acetate	[152]
HSD17B4	Metabolism of androgen	rs7737181	iSNP	5	Abiraterone acetate	[145]
AKR1C3	Metabolism of androgen	rs12529	cSNP	10	Abiraterone acetate	[157]
ABCB1	Encodes protein act as drug excretion pump	rs2032582	cSNP	7	Docetaxel + Thalidomide	[158]
		rs1128503				
		rs1045642				
ABCB11	encodes protein act as drug excretion pump	rs7602171	iSNP	2	Docetaxel + Thalidomide	[159]
ABCG2	encodes protein act as drug excretion pump	rs2231142	cSNP	4	Docetaxel + Vinorelbine/Estramustine phosphate	[160]
SLCO1B3	Androgen transporter	rs4149117	cSNP	12	Docetaxel	[161]
SLCO2B1	Encodes protein act as androgen transporter	rs1077858	iSNP	11	Docetaxel	[162]
		rs1789693	iSNP			[163]
		rs12422149	cSNP			[164]
GNRH2	Related to the synthesis of androgen	rs6051545	cSNP	20	Abiraterone acetate	[165]
SHBG	Androgen-binding protein	rs6259	cSNP	17	Enzalutamide	[150]
AR	Steroid receptor	CAG repeat	Coding region	Xq11-12	Docetaxel	[166]
ATP7A	Copper level regulator	rs2227291	SNV	X	Cisplatin	[167]
ABCC6	Transporter protein	rs2238472	SNV	16	Docetaxel + Thalidomide	[168]
ABCB4	MRP6	rs2302387	SNV	7	Docetaxel + Thalidomide	[169]
ESR1	Steroid receptor	rs1062577	rSNP	6	Docetaxel + Thalidomide	[152, 170]
		rs2234693	iSNP			
		rs9340799				
NR3C2	Steroid receptor	rs5522	cSNP	4	Docetaxel + Thalidomide	[165]
YB-1	Transcription factor	rs12030724	iSNP	1	Abiraterone	[171]
HIF1A	Transcription factor	rs11549465	cSNP	14	Docetaxel	[171]
ARRDC3	Target gene of AR	rs2939244	rSNP	5	Abiraterone	[149]
FLT1	Androgen-binding	rs9508016	rSNP	13	Enzalutamide	[149]
SKAP1	Protein steroid receptor	rs6054145	rSNP	20	Abiraterone acetate	[149]
FBXO32	Steroid receptor	rs7830622	rSNP	8	Abiraterone acetate	[67]
BNC2	Steroid RECEPTOR	rs16934641	rSNP	9	Abiraterone acetate	[149]
TACC2	Transcription	rs3763763	rSNP	10	Bicalutamide	[149]
ALPK1	Factor	rs2051778	rSNP	4	Enzalutamide	[172]
LSAMP	Transcription	rs13088089	rSNP	3	Abiraterone	[173]
CCL17	Transcription	rs13088089	rSNP	3	Leuprolide	[173]
ALPK1	Transcription factor	rs2051778	rSNP	4	Enzalutamide	[174]
LSAMP	Transcription	rs13088089	rSNP	3	Bicalutamide	[175]
NAT2	Xenobiotics detoxifier	rs1799931	SNV	8	Docetaxel + Thalidomide	[176]
PSMD7	NFκB targeted gene	rs2387084	rSNP	16	Enzalutamide	[173]
PPAR-δ	Fatty acid uptake, transport and β-oxidation	rs6922548	SNP	6	Docetaxel + Thalidomide	[177]
		rs2016520				[178]
		rs1883322				[178]
		rs3734254				[179]
		rs7769719				[168]
		rs4148943				[168]
MON1B	NFκB targeted gene	rs284924	rSNP	16	Abiraterone acetate	[67]
GSTM3	Antioxidant	rs7483	cSNP	1	Docetaxel + Thalidomide	[180]
GSTP1	Antioxidant	rs1138272	cSNP	11	Docetaxel + Thalidomide	[126]
CAT	Antioxidant	rs564250	gSNP	11	Docetaxel + Thalidomide	[159]
CHST3	Development and maintenance of the skeleton	rs12418	SNV	10	Docetaxel + Thalidomide	[160]
		rs730720				
		rs4148950				
		rs1871450				
		rs4148945				
SLC28A3	Nucleoside transporter	rs56350726	cSNP	9	Docetaxel + Thalidomide	[161]
SLC5A6	Transporter	rs1395	cSNP	2	Docetaxel + Thalidomide	[159]
SLC10A2	Sodium/bile acid Co-transporter	rs2301159	SNV	13	Leuprolide	[176]
SULT1C2	Encode Sulfotransferase 1C2 in humans	rs1402467	SNP	2	Docetaxel + Thalidomide	[]
LRP2	Encode protein act as a transporter for Sterol and Steroid	rs6433107	iSNP	2	Abiraterone acetate	[182]
		rs3944004				
		rs830994				
		rs3770613				
		rs831003				
EGF	Growth factor	rs4444903	rSNP	4	Degarelix	[183]
IRS2	Growth factor	rs7986346	gSNP	13	Degarelix	[149]
TGFBR2	TGF-β signaling	rs3087465	iSNP	3	Abiraterone acetate	[184]
BMP5	TGF-β signaling	rs317027	gSNP	4	Abiraterone acetate	[172]
IL18	Cytokine	rs187238	rSNP	11	Docetaxel + Thalidomide	[185]
APC	Wnt signaling	rs2707765	iSNP	5	Degarelix	[186]
		rs497844				
BGLAP	Metabolism of bone	rs1800247	rSNP	1	Estramustine phosphate	[187]
EDN1	Vasoconstrictor	rs1800541	iSNP	6	Enzalutamide	[188]
		rs2070699				
CASP3	Apoptosis	rs4862396	gSNP	4	Abiraterone acetate	[149]
TRMT11	Methyltransferase	rs1268121	iSNP	6	Estramustine phosphate	[189]
		rs6900796				
COMT	Methyltransferase	rs4680	cSNP	22	Estramustine phosphate	[190]
KIF3C	miRNA target site	rs6728684	rSNP	2	Docetaxel + Thalidomide	[191]
IFI30	miRNA target site	rs1045747	rSNP	19	Docetaxel + Thalidomide	[191]
CDON	miRNA target site	rs3737336	rSNP	11	Docetaxel + Thalidomide	[192]
GABRA1	miRNA target site	rs998754	rSNP	5	Docetaxel + Thalidomide	[67]
PALLD	miRNA target site	rs1071738	rSNP	4	Abiraterone acetate	[191]
VEGFA	Angiogenesis	rs1570360	rSNP	6	Docetaxel, Celecoxib + Cyclophosphamide	[193]
SYT9	miRNA target site	rs4351800	rSNP	11	Abiraterone acetate	[194]
		rs16901979,	gSNP			
		rs7931342				

Genome-wide association studies reported that androgen metabolism and pharmacotherapy outcomes are related to multiple SNPs reported in various genes. For example, a variant cSNP (rs1047303) of 3β-hydroxysteroid dehydrogenase 1 (3β-HSD1) encodes by *HSD3B1* influences the enzymatic activity significantly, so carriers of this variant are the poor drug metabolizer [141]. The prognostic impact of this variant was observed in patients in the USA and confirmed in an Asian cohort study, which reported that this variant is rare in Asian patients but successfully validated as a prognostic marker in primary ADT plus docetaxel for HSPC [142]. There are four reported SNPs (rs6162, rs743572, rs1004467, and rs6163) in the CYP17A1 genes influencing prostate cancer progression to CRPC after ADT. Furthermore, the risk of development of CRPC is also linked with dehydroepiandrosterone (DHEA), a steroidal hormone that acts as a precursor of intratumoral androgen biosynthesis that controls the progression of cancer and is an important target for novel therapies [143]. Enzymes encoded by CYP19A1 catalyze the conversion of androgens to estrogen. Three SNPs (rs10459592, rs2470152, rs4775936) reported in this gene are related to the risk of development of prostate cancer [144]. In *HSD3B1*, the validation of prognostic values of cSNP (rs1047303 and rs1856888) was performed in ADT plus docetaxel therapy for HSPC, and it was found that the low risk of progression of the disease is linked with (rs1856888) which is located in the variant G allele [145]. Another study on iSNP in *CYP19A1* described that a high risk of progression of the disease is associated with the variant C allele in rs1870050 [146]. The function and expression pattern of *HSD17B2* is reported to reduce prostate cancer, as it suppresses AR signaling and cell growth by blocking androgen synthesis. Various studies on gene expression profiling have explained that the disease progression is caused by altering expression patterns of specific genes (*HSD17B2, HSD17B3, SRD5A1,* and *SHBG*) [147]. *SLCO2B1* and *SLCO1B3* are involved in the steroidal hormone uptake, and thrombotic thrombocytopenic purpura (TTP) is linked with three SNPs present in *SLCO2B1* expressed in various tissues. These SNPs transport steroid conjugates, such as estrone-3-sulfate and DHEAS. *SLCO1B3* expresses in different types of cancer cells and is responsible for the uptake of several hormones [148]. *In-vivo* studies confirmed that tumor growth is enhanced by HIF1a signaling, whereas its stable expression is linked with the restoration of tumor growth. After evaluating SNPs in the binding sites of estrogen and androgen receptors, it was found that the 5 SNPs localized on *ARRDC3, TACC2, SKAP1, FLT1,* and *BNC2* are specifically associated with prostate cancer mortality [149]. It is also observed that *BNC2* (rs16934641) is linked with the progression of the disease, while *ALPK1* (rs2051778) is associated with ACM. SNP in the *TACC2* (rs3763763) is involved in ACM and prostate cancer-specific mortality. The less significant associations of *SKAP1* (rs7209855) and *KLHL14* (rs12970312) were observed with PCSM [150]. Similarly, *NR4A2* (rs2691786), *FBXO32* (rs7830622), *AATF* (rs 9330247), and *KLHL14* (rs12970312) were found to be less significantly associated with ACM. The high expression level of *BGLAP* is responsible for the survival of bone metastasized tumor cells in prostate cancer [151] (Table 2).

**Fig. 3:**
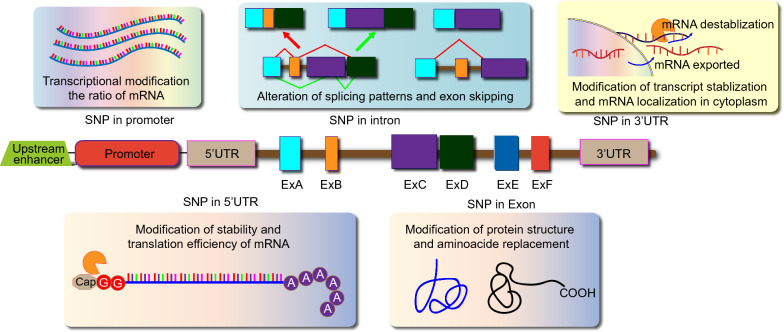
Types, functions, and locations of single-nucleotide polymorphisms (SNPs) in translated and untranslated regions of genes

The survival rate of CRPC patients has been improved with the use of novel androgen receptor pathway inhibitors (ARPIs) (Enzalutamide, Apalutamide, Darolutamide, and Abiraterone) because their therapeutic effects depend upon the activity of molecules involved in their uptake and metabolism. For instance, *SLCO2B1* encodes OATP2B1, which is responsible for the uptake of abiraterone into cells metabolized by 3-HSD and 5-reductase [195]. It is reported that the therapeutic effect of abiraterone depends on SNPs in the genes involved in the transport and metabolism of androgen. Few SNPs are associated with prognosis after ARPI treatment, such as (rs2486758) in *CYP17A1* and (rs1047303) in *HSD3B1*. The overlapping of several genes has been observed in the prognosis because ARPIs and primary ADT outcomes depend on the SNPs located in different genes (*CYP17A1* and *YB-1*) [61]. Similarly, SNPs in various genes [(rs1789693, rs1077858, and rs12422149 in *SLCO2B1*), (rs523349 in *SRD5A2*) and (rs1047303 in *HSD3B1*)] are acting as prognostic markers in ARPIs for CRPC and primary ADT for HSPC. It has been found that a variant allele in *HSD3B1* (rs1047303) is a prognostic marker for patients treated with abiraterone [196]. Docetaxel is used to treat various types of cancers. Several studies have shown a correlation between efficacy and adverse effects of docetaxel with genetic polymorphism in transport genes (*ABCC2*, *ABCB1*, *ABCG1*, *SLCO1B3, ABCG2*) and metabolizing genes (*CYP1B1*, *CYP3A4*, *CYP2C8,* and *CYP3A5*). In *CYP1B1,* reported cSNP (rs1056836, 4326C>G, L432V) is linked with the poor therapeutic response of the drug and prognosis [197]. The patient's response to taxane chemotherapy depends on SNPs found in various positions in estrogen receptor-1 (ESR1). These SNPs serve as potential predictive biomarkers for taxane chemotherapy. The resistance to Taxane therapy is induced by the OATP1B3 transport protein in prostate cancer cells encoded by *SLCO1B3* [198]. Another predictive marker for PCa that affects the efficacy of taxane therapy is cSNP (rs4149117), located in *SLCO1B3*. Although SNPs are influencing pharmacotherapy, but still there are only a few genetic markers that have been used in pharmacotherapy or individualized treatment strategy for cancer patients [199]. In markers validation studies, the reproducibility of some SNPs has occurred successfully. In contrast, other studies failed to produce consistent results because of racial differences, and there are variations in the frequency of genetic polymorphisms [200]. Figure 3 exhibits the signals transduction through AR.

## Conclusion

Disease risk is associated with genetic variations. Most PCa research focuses on a limited number of genetic markers commonly used in clinical practice. These markers include PSA, TMPRSS2-ERG Gene Fusion, PTEN Loss, and mutations in BRCA1 and BRCA2. However, many novel genetic markers have been identified in recent years. Genome-wide association studies (GWASs) provide valuable information on identifying SNP groups that accurately predict prostate cancer risk, development, and pharmacotherapy response. Clinically, multiple drugs are available to treat Prostate Cancer, but Individualized treatment regimens for patients with advanced-stage prostate cancer are largely determined by the availability of suitable genetic biomarkers (SNPs). Combining SNPs with traditional clinicopathological parameters will lead to earlier diagnosis, better prognoses, and more effective pharmacotherapy. Additionally, SNP-based personalized medicine will reduce the need for ineffective pharmacotherapy trials in prostate cancer patients. Further studies are needed to validate these SNPs in PCa progression and to identify biomarker inter-individual variations. In terms of the future perspective of this field, integrating multiple genetic markers, along with clinical and pathological parameters, may enhance risk stratification, prognosis prediction, and treatment selection. This will also help tailor interventions and healthcare decisions based on individual genetic makeup.

## Data Availability

Not applicable.
